# Digital holographic microscopy based on a modified lateral shearing interferometer for three-dimensional visual inspection of nanoscale defects on transparent objects

**DOI:** 10.1186/1556-276X-9-471

**Published:** 2014-09-04

**Authors:** Kwang-Beom Seo, Byung-Mok Kim, Eun-Soo Kim

**Affiliations:** 1 HoloDigilog Human Media Research Center (HoloDigilog), 3D Display Research Center (3DRC), Kwangwoon University, 447-1 Wolge-Dong, Nowon-Gu, Seoul 139-701, Korea

**Keywords:** Lateral shearing interferometer, Digital holographic microscopy, Defect detection of transparent materials, Three-dimensional visual inspection, Depth and phase measurement and metrology

## Abstract

A new type of digital holographic microscopy based on a modified lateral shearing interferometer (LSI) is proposed for the detection of micrometer- or nanometer-scale defects on transparent target objects. The LSI is an attractive interferometric test technique because of its simple configuration, but it suffers from the so-called 'duplicate image’ problem, which originates from the interference of two sheared object beams. In order to overcome this problem, a modified LSI system, which employs a new concept of subdivided two-beam interference (STBI), is proposed. In this proposed method, an object beam passing through a target object is controlled and divided into two areas with and without object information, which are called half-object and half-reference beams, respectively. Then, these two half-beams make an interference pattern just like most two-beam interferometers. Successful experiments with a test glass panel for mobile displays confirm the feasibility of the proposed method and suggest the possibility of its practical application to the visual inspection of micrometer- or nanometer-scale defects on transparent objects.

## Background

Digital holographic microscopy (DHM) is a technique which enables the numerical reconstruction of recorded holograms with a computer [1,2]. In DHM, amplitude and phase information of a target object is recorded on a charge-coupled device (CCD) camera as an interference pattern, which is called hologram, through an objective lens. Then, similar to an optical holography, the diffraction of wavefronts from the recoded hologram is computed and numerically propagated along the reconstruction distance to any chosen observation plane with a computer system. Thus, based on this numerical knowledge of the propagated complex wavefront, the amplitude and the phase of a target object can be computed, which can lead to micrometer- or nanometer-scale quantitative measurements of the target object [3-6].

When investigating, DHM obtains the depth information of a specimen and resolves phase differences corresponding to depth differences as price as several nanometers. Thus, it would be well suited for detection of nanometer- or micrometer-scale defects on transparent objects [7-9]. Furthermore, since the phase information provided by DHM allows us to visualize the detailed topological shapes of those defects, DHM attracts the attentions of various information technology industries [10].

In general, DHM can be implemented with an interferometer system. There are two kinds of interferometer systems which are two-beam and single-beam interferometers, which include the Michelson and the Mach-Zehnder interferometers and shearing interferometer, respectively [1-6]. Here, the recorded hologram by DHM is usually composed of three components which are DC bias and real and virtual images, in which DC bias and virtual image are undesired terms in the numerical reconstruction process. Thus, these terms need to be eliminated because their elimination results in an enhancement of image quality.

Kim et al. suggested a modified Cochran interferometer for removing DC bias and virtual image by using a phase shifting method [11]. Cuche et al. also proposed a Fresnel diffraction method for removing DC bias and virtual image in the frequency domain with a low-pass filter [1]. Based on these interferometers, various DHM systems have been proposed for defect detection on transparent materials. Xu et al. presented an in-line DHM system for testing micro-structures with a long-distance microscope [12]. Schulze et al. also proposed another DHM system based on a modified Mach-Zehnder interferometer to detect defects on semiconductor wafers [9]. Kuhn et al. obtained 3-D information of the transparent microstructure from the depth data detected by DHM [6]. In addition, Wahba and Kreis measured the refractive index of optical fibers by DHM [13], and Colomb et al. proposed a digital holographic reflectometry based on a modified Mach-Zehnder interferometer [14].

In addition, a single-beam lateral shearing interferometer (LSI) has been also applied for DHM because of its simple optical configuration. Sen and Puntambekar suggested shearing interferometers for prism testing under manufacturing [15]. Nyyssonen et al. presented a simple wavefront shearing interferometer for lens testing [16]. In addition, Merzkirch generally analyzed the shearing interferometer [17]. Matsuda et al. suggested a holographic LSI system for the real-time measurement of large liquid surface deformations [18]. Moreover, Choi et al. presented wedge-plate shearing interferometers for collimating testing of laser beam using a moiré technique [19]. In addition, Singh et al. suggested a LSI-based DHM system for imaging small biological specimens [20].

In most LSI systems, hologram patterns are formed by two laterally sheared object beams. This is unlike the conventional two-beam interferometer, where a hologram pattern is generated by the interference of two separate object and reference beams. In other words, the conventional LSI system has no obvious reference beam. Thus, the recorded hologram inevitably includes not only DC bias and twin images like in most two-beam interferometers but also undesired images, so-called duplicate images which result from the two sheared object beams getting involved in interference. This deteriorates the operational performance of the conventional LSI system.

In order to alleviate this drawback, in this paper, a modified LSI system employing a new concept of subdivided two-beam interference (STBI) is proposed, and several key parameters for this proposed system are derived based on ray optics. Here, the object beam passing through a transparent target is divided into two areas with and without object information, which is called half-object and half-reference beams, respectively. Thus, these two half-beams make an interference pattern without the aforementioned duplicate images [21]. Additionally, a new type of DHM system based on this modified LSI is also implemented for visual inspection of micrometer- or nanometer-scale defects on transparent objects. For confirmation of the feasibility of the proposed method, experiments with a simple touch-glass panel for mobile displays are carried out, and the results are comparatively analyzed with those of the conventional method in terms of the effects of duplicated images.

## Methods

### Modified lateral shearing interferometer

**Figure 1:**
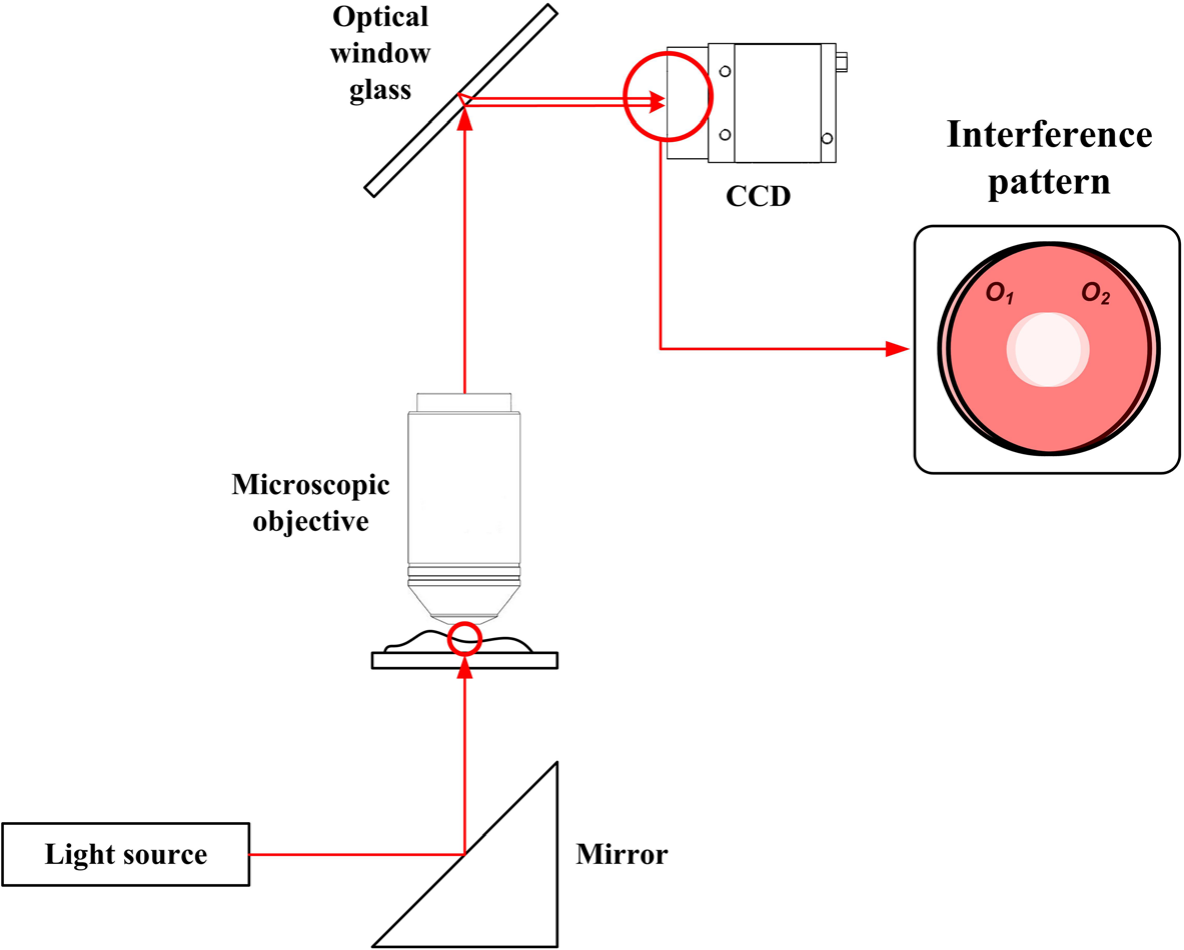
Configuration of the conventional LSI system.

**Figure 2:**
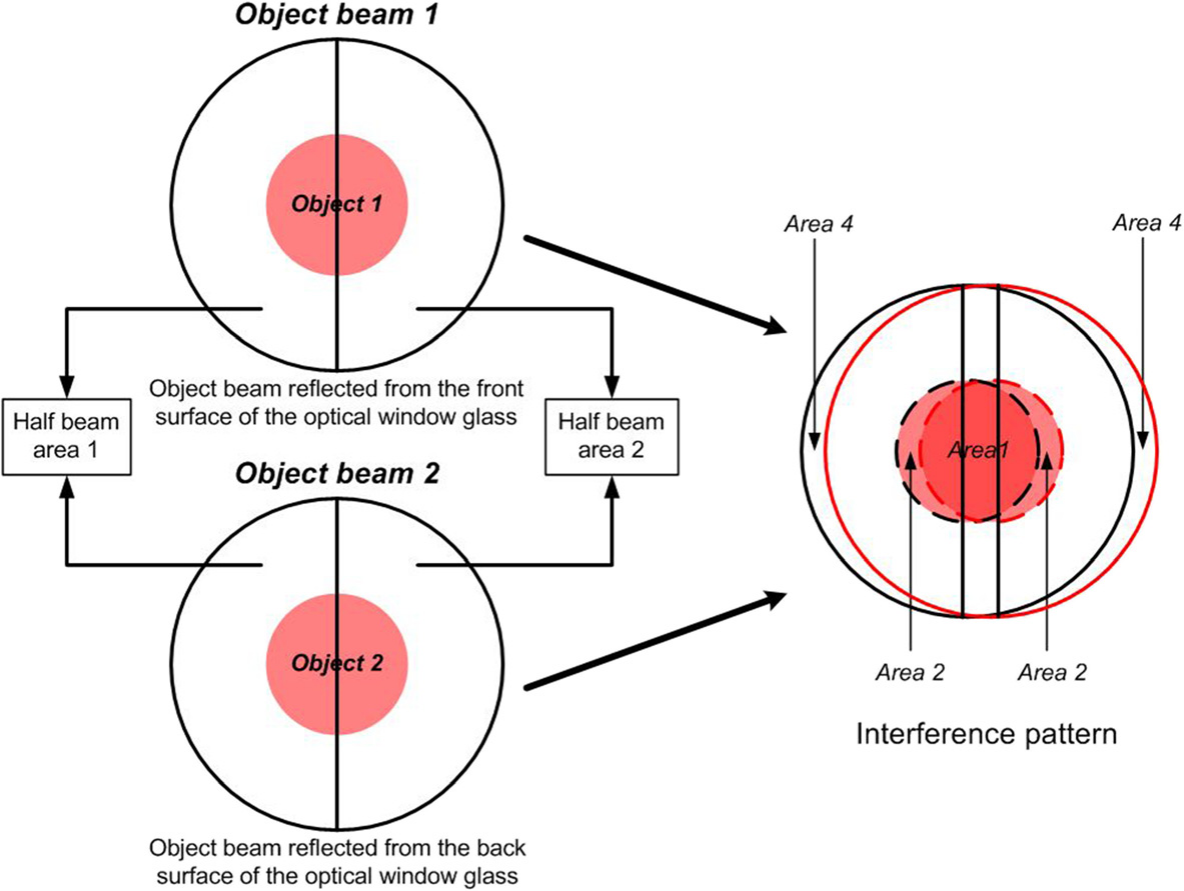
Detailed description of the interference pattern generated in the conventional LSI system.

In the conventional LSI system, a hologram pattern is formed by the difference of the optical path length in two object beams caused by the thickness of an optical window glass. Thus, the spatial period and beam area of the hologram pattern are determined by the thickness of an optical window glass. As shown in Figure 1, an object image is usually magnified by using an objective lens in the DHM system, and the enlarged object beam is reflected from front and back surfaces of an optical window glass. Then, these two reflected object beams make an interference pattern, and it is recorded on a CCD camera. Here, the recorded hologram contains duplicate images, which are uniquely generated in the conventional LSI system due to the interference of two sheared object beams, as well as DC bias and twin images. These duplicate images actually degrade the reconstructed real image and make it hard to extract the depth information of a target object from the recorded hologram.Here, if a specimen under investigation is transparent, each object beam reflected from front and back surfaces of an optical window glass can be modeled as a mixture of two subdivided object beams with and without object information as shown in Figure 2. That is, each object beam is subdivided into two parts such as one inner part containing object data, called half-object beam, and the other outer part containing no object data, called half-reference beam. Thus, these two sets of two subdivided object beams interfere with each other and make a hologram pattern in the conventional LSI system.

Here, the intensity of this interference pattern recorded on the CCD camera can be expressed by Equation 1:

(1) $$\begin{array}{ll} I\left(x,y\right) & ={\left|O_{F}\left(x,y\right)+O_{B}\left(x,y\right)\right|}^{2}=\left|\left\{O_{1}\left(x,y\right)+R_{1}\left(x,y\right)\right\}\right)\\ & \;{\left(+\left\{O_{2}\left(x,y\right)+R_{2}\left(x,y\right)\right\}\right|}^{2}\\ & =\left[\left\{{I_{O_{1}}}^{2}+{I_{R_{1}}}^{2}\right\}+\left\{{I_{O_{2}}}^{2}+{I_{R_{2}}}^{2}\right\}\right]\\ & \;+\left[\left(R_{1}+R_{2}\right)\left({O_{1}}^{*}+{O_{2}}^{*}\right)+\left(O_{1}+O_{2}\right)\left({R_{1}}^{*}+{R_{2}}^{*}\right)\right]\\ & \;+\left[{O_{1}}^{*}O_{2}+{O_{2}}^{*}O_{1}\right]+\left[{R_{1}}^{*}R_{2}+{R_{2}}^{*}R_{1}\right] \end{array}$$

where *O*_*F*_ denotes the object beam reflected from the front surface of an optical window glass, which is assumed to be composed of *O*_1_ and *R*_1_ with and without object information, respectively. Likewise, the object beam reflected from the back surface of an optical window glass is denoted as *O*_*B*_, which is also assumed to consist of *O*_2_ and *R*_2_. In addition, $I_{O_{1}}$, $I_{R_{1}}$, $I_{O_{2}}$ and $I_{R_{2}}$ represent the intensities of *O*_1_, *R*_1_, *O*_2_ and *R*_2_, respectively.As seen in Equation 1, the first and second square brackets in Equation 1 represent the DC bias and the twin (real and virtual) images, respectively. Here, the second term in Equation 1 is referred to as 'Area 2’ in Figure 2. These two terms look very similar with those of most two-beam interferometers. However, the third term in Equation 1, which is referred to as 'Area 1’ in Figure 2, represents undesired images, which are called duplicate images. These may be generated only in the LSI system due to the interference of two sheared object beams. Since the 'Area 1’ in Figure 2, which is referred to as the third term, is partially overlapped with those of twin images, these images are excessively reconstructed together with twin images. Thus, these images must be removed because they degrade the quality of reconstructed images.

**Figure 3:**
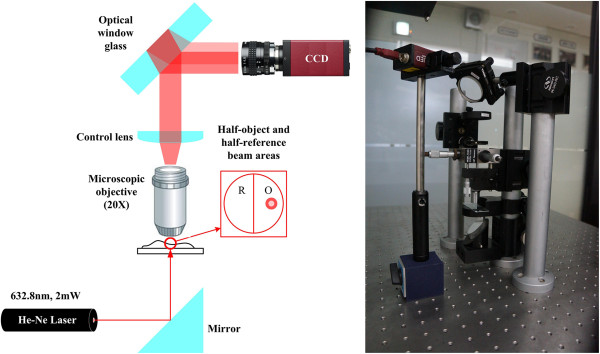
Experimental setup for the proposed LSI-based DHM system.

Here, the DC bias and the virtual image can be spatially filtered out in the Fourier domain by using a low-pass filter just like in most two-beam interferometers. However, duplicate images cannot be separated from the reconstructed images because these are partially overlapped with twin images. Thus, to solve this problem, an obvious reference beam without object information is needed. However, this reference beam cannot be obtained in the conventional LSI system because of its single beam-based operational property. Accordingly, in this paper, a modified LSI system to generate an obvious reference beam from the single beam based on a new concept of STBI is proposed for removing those duplicate images.In the proposed system as shown in Figure 3, the object beam passing through a target object and being magnified by an objective lens is divided into two areas with and without object information, which is called half-object and half-reference beams, respectively, by controlling the object's location, incident object-beam angle, and thickness of an employed optical glass window. After being reflected from the front and back surfaces of the optical window glass, two sheared object beams, in which each object beam is composed of half-object and half-reference beams, are generated. Then, two sets of half-object and half-reference beams of two object beams make an interference pattern without duplicate images.

### Hologram formation with half-object and half-reference beams

**Figure 4:**
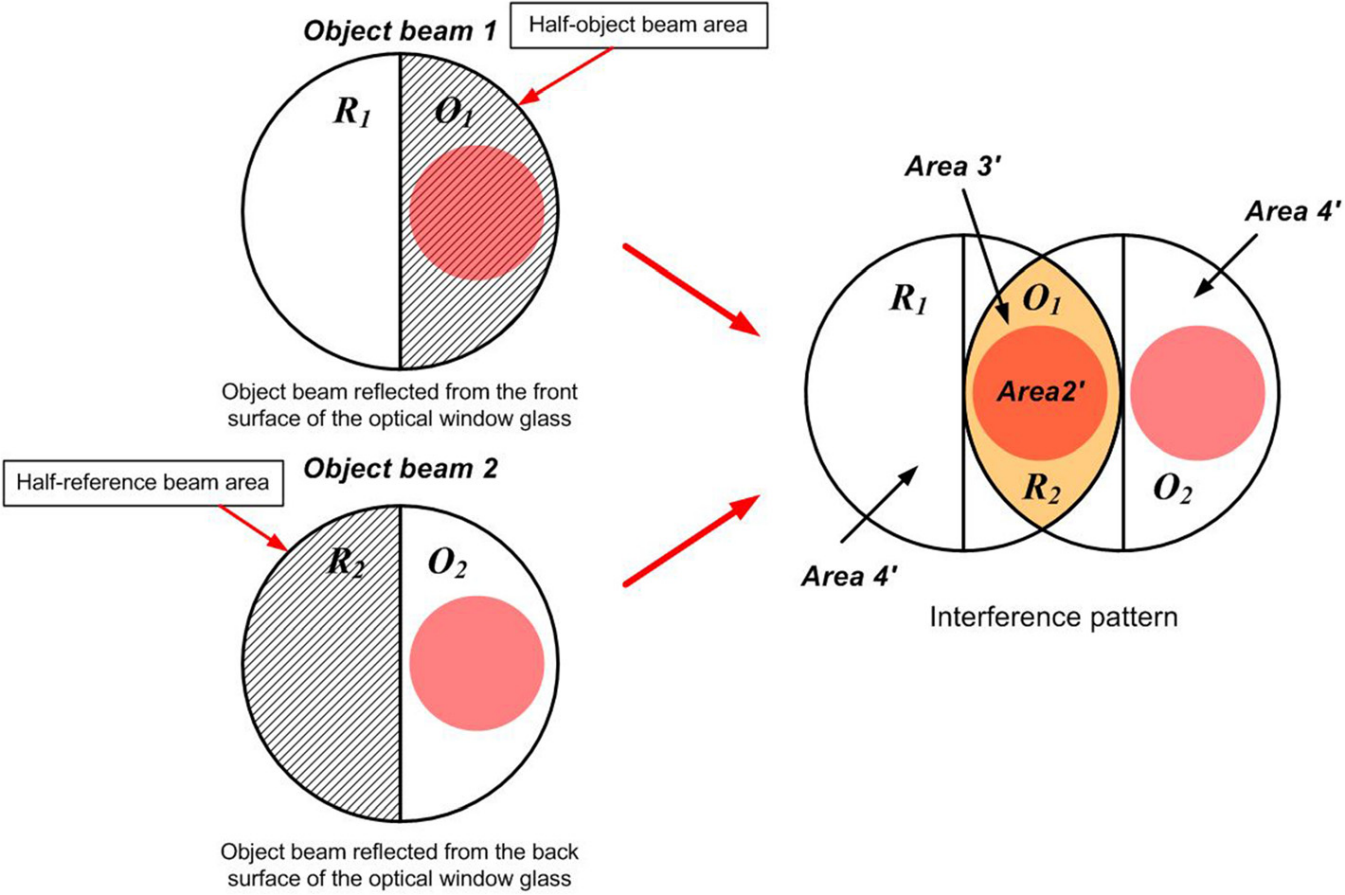
**A new concept of subdivided two**-**beam interference.**

Figure 4 shows a new concept of STBI for implementing the modified LSI system. For the proposed system to be applicable to DHM-based defect detection, location of a target object, thickness and refractive index of an employed optical window glass, and the incident object-beam angle to the optical window glass must be carefully controlled. That is, for subdividing a single object beam into two half-object and half-reference beams with and without object information, respectively, the target object should be located not right on the optic axis just like in the conventional LSI system but on the side location somewhat displaced from the optic axis.

As shown in Figure 4, the object and reference beam areas of *O*_1_ and *R*_1_ with and without object information, respectively, are reflected from the front surface of the optical window glass. In addition, another object and reference beam areas of *O*_2_ and *R*_2_ are also reflected from the back surface of the optical window glass. Thus, a hologram pattern can be partially formed between the object-beam area *O*_1_ and the reference-beam area *R*_2_ just like in most two-beam interferometers.

As mentioned above, in the proposed method, *O*_1_ and *R*_2_ make an interference pattern, which is composed of two areas including 'Area 2′’ and 'Area 3′’ as shown in Figure 4. Here, the 'Area 2′’ and the 'Area 3′’ represent twin (real and virtual) images and DC bias, respectively, just like those of the conventional two-beam interferometer.

#### Lateral shearing distance by two object beams

**Figure 5:**
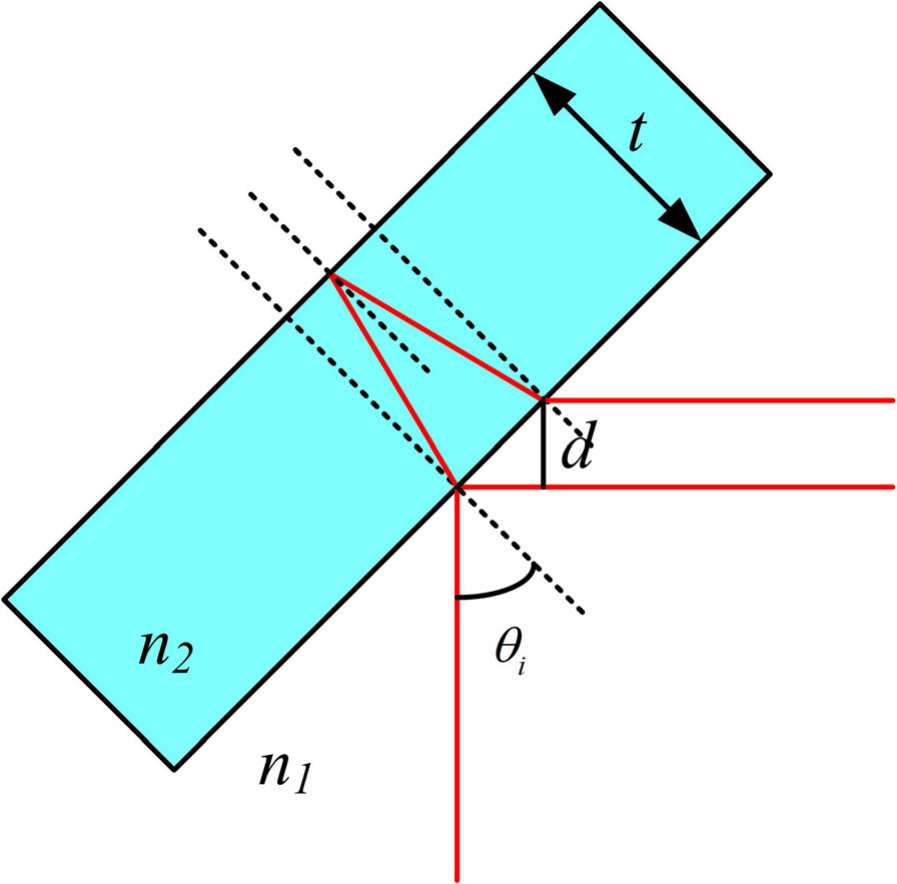
Lateral shearing distance between two object beams.

In the proposed system, the hologram pattern is formed by two sheared object beams, which are reflected from the front and back surfaces of an optical window glass. In particular, in the conventional LSI system, this hologram unavoidably contains undesired duplicate images due to the short lateral shearing distance (LSD) between two object beams, which mean more than half of each object beam is overlapped together. However, in the proposed system, this problem can be alleviated by controlling the LSD by using the incident beam angle to the optical window glass as well as the thickness and refractive index of the optical window glass as shown in Figure 5.

The proposed LSI system based on a new concept of STBI can be regarded as a two-beam interferometer. Here, the LSD, *d*, can be derived from a trigonometric relationship among incident beam angle, thickness, and refractive index of the optical window glass as shown in Equation 2:

(2) $$d=2t\text{cos}\theta_{i}\text{tan}\left({\text{sin}}^{-1}\left(\frac{n_{1}}{n_{2}}\text{sin}\theta_{i}\right)\right)$$

where *t* and *θ*_*i*_ denote the thickness of an optical window glass and the incident angle of the object beam onto the optical window glass, respectively. Also, *n*_1_ and *n*_2_ represent the refractive index in the air and in the optical window glass, respectively.

#### Effective interference area

**Figure 6:**
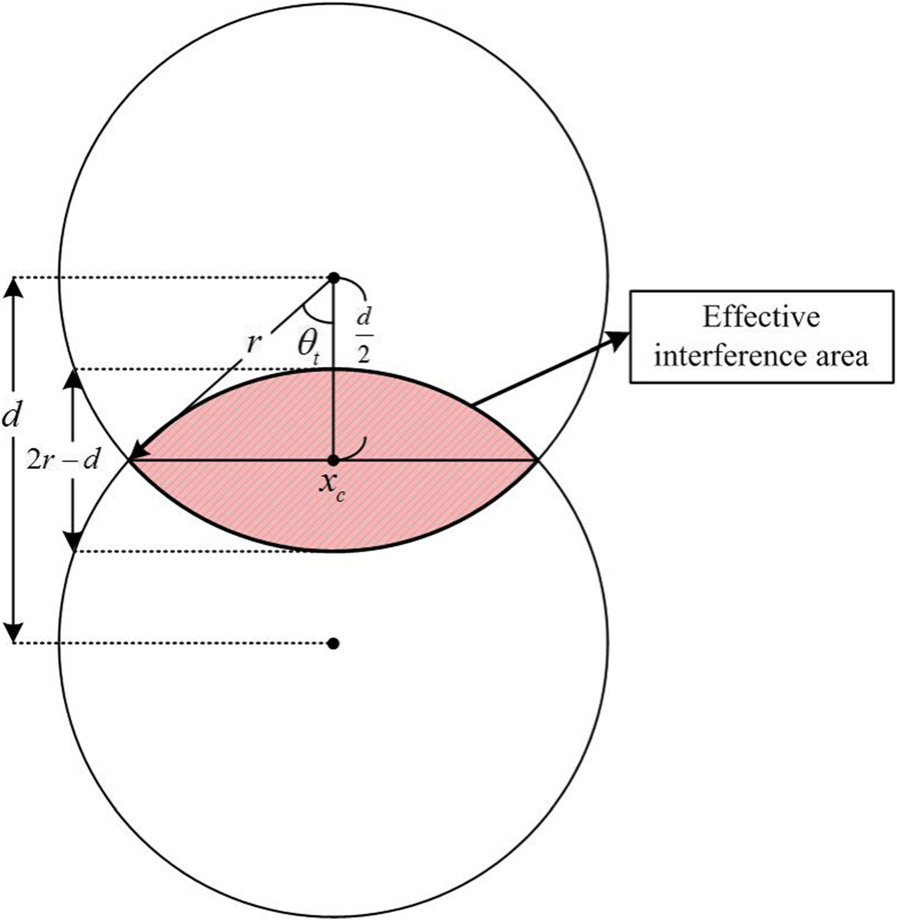
An EIA with two half-object and half-reference beams.

In Figure 6, the angle (*θ*_*t*_) is defined as the two-beam intersection angle (TIA) to make an interference pattern between two sheared object beams without duplicate images. This *θ*_*t*_ value can be derived from a trigonometric relationship between two parameters of object beam's radius of *r* and LSD of *d*, which is given by Equation 3.

(3) $$\theta_{t}=\text{arc}\;\text{cos}\left(\frac{d}{2r}\right)$$

According to Equation 3, *θ*_*t*_ is calculated to range from 0° to 60° as shown in Figure 6. Of course, the optimum value of *θ*_*t*_ becomes 60°, in which two halves of each object beam can interfere in the largest beam areas. However, if *θ*_*t*_ is larger than 60°, duplicate images begin to generate because more than the halves of each object beam are overlapped. Thus, *θ*_*t*_ must be controlled to be 60° in the proposed LSI system.

Base on the TIA, the size of the effective interference area (EIA) *S*_eff_, in which a meaningful interference pattern is formed, can be calculated by Equation 4:

(4) $$S_{\text{eff}}=4\pi r^{2}\theta_{t}-\mathrm{rd}\text{sin}\theta_{t}$$

when *θ*_*t*_ is 60°, just half-object and half-reference beams can be overlapped to make a hologram. Under this condition, the EIA is maximized. In addition, the optimized location of the target object of *x*_*c*_ in the object plane can be determined by Equation 5 as seen in Figure 6:

(5) $$x_{c}=\frac{2r-d}{2}$$

In the conventional LSI system, the LSD is estimated to be in the range of 0 to 0.76 mm depending on the incident angle *θ*_i_, which varies from 0° to 90° when the thickness of the optical window glass and the radius of the object beam are given by 1 and 5 mm, respectively. Then, from Equation 3, *θ*_*t*_ is calculated to range from 85.72° to 90°, which means that the EIA may be much reduced.

On the other hand, in the proposed method, the LSD is calculated to be in the range of 0 to 9 mm in case the thickness of the optical window glass and the radius of the object beam are 12 and 5 mm, respectively. Moreover, *θ*_*t*_ is ranged from 23° to 90°, and the EIA is calculated to be about 3.07 × 10^-4^ mm^2^ for *θ*_*t*_ = 60°.

### Digital reconstruction of the recorded hologram pattern

The hologram pattern recorded by the proposed system of Figure 3 contains both intensity and phase information of a target object. Thus, from this hologram pattern, phase data of the target object can be extracted, and a 3-D image can be numerically reconstructed with the quantitative phase contrast method [22].

For extracting depth data of a target object, Fresnel diffraction approximation and the angular spectrum method are mostly used [23]. Here, in this paper, the recorded hologram is transformed into the image plane domain by using the angular spectrum method and then the complex amplitude on the image plane is converted into the phase data. With this phase data, the 3-D object image is numerically reconstructed.

Basically, in the proposed LSI system, the hologram pattern of a target object is formed by interfering between two half-object and half-reference beams. Thus, the intensity of the interference pattern on the hologram plane is given by Equation 6:

(6) Ix,y=O1x,y+R2x,y2=O12+R22+O1*R2+O1R2*

For numerical reconstruction, another reference beam *R*_2_ illuminates the recorded hologram pattern of Equation 7. Then, the complex amplitude on the hologram plane is given by as follows:

(7) $$U\left(x,y,0\right)=I\left(x,y\right)R_{2}\left(x,y\right)$$

Here, the angular spectrum of this complex amplitude is given by Equation 8:

(8) $$\hat{U}\left(f_{x},f_{y},0\right)=\int \int_{-\infty }^{\infty }U\left(x,y,0\right)\text{exp}\left\{-j2\pi \left(f_{x}x+f_{y}y\right)\right\}\text{dxdy}$$

Where *f*_x_ and *f*_y_ denote the spatial frequencies in the *x*- and *y*-direction, respectively. By applying a low-pass filtering operation to this angular spectrum, the DC bias and the virtual image can be removed.

(9) $${\hat{U}}_{L}\left(f_{x},f_{y},0\right)=\sum_{n=1}^{L}\sum_{m=1}^{L}\delta \left(f_{x}-f_{x_{n}},f_{y}-f_{y_{m}},0\right)\hat{U}\left(f_{x},f_{y},0\right)$$

Where $f_{x_{n}}$ and $f_{y_{m}}$ represent the filtered spatial frequencies in the *x*- and *y*-direction, respectively. By using the delta-function, the real object image can be filtered out in these spatial frequencies ranges. ${\hat{U}}_{L}\left(f_{x},\;f_{y},\;0\right)$ means the filtered complex amplitude of the real image in the spatial frequency domain. Thus, the 3-D image can be reconstructed using the angular spectrum method as follows.

(10) $${\hat{U}}_{L}\left(f_{x},f_{y},d\right)={\hat{U}}_{L}\left(f_{x},f_{y},0\right)\text{exp}\left\{\mathrm{ik}\sqrt{1-\lambda^{2}{f_{x}}^{2}-\lambda^{2}{f_{y}}^{2}}d\right\}$$

where *k*, *λ*, and *d* denote the wave number, the wavelength of a light source, and the reconstruction distance, respectively. Here, the filtered complex amplitude in the frequency domain is converted into the complex amplitude in the spatial domain by inverse Fourier transformation:

(11) $$U\left(x,y,d\right)=\int \int_{-\infty }^{\infty }{\hat{U}}_{L}\left(f_{x},f_{y},d\right)\text{exp}\left\{j2\pi \left(f_{x}x+f_{y}y\right)\right\}df_{x}df_{y}$$

Now, the complex amplitude on the hologram plane is converted into that on the image plane. That is, the intensity *I*(*x*, *y*, *d*) of the complex amplitude *U*(*x*, *y*, *d*) on the image plane is given by as follows:

(12) $$I\left(x,\;y,\;d\right)={\left|U\left(x,\;y,\;d\right)\right|}^{2}$$

The phase data of the target object which is located at the distance *d* can be obtained from the real and imaginary parts of the complex amplitude on the image plane:

(13) ϕx,y,d=arctanImUx,y,dReUx,y,d

The phases are individually computed from the complex amplitude of the two states of *ϕ*_*O*_(*x*, *y*, *d*) with the object and *ϕ*_*R*_(*x*, *y*, *d*) without the object. Thus, *U*_*O*_(*x*, *y*, *d*) represents the complex amplitude with the object information, while *U*_*R*_(*x*, *y*, *d*) means the complex amplitude without the object information. Each phase data of those complex amplitudes is given by Equations 14 and 15, respectively:

(14) ϕOx,y,d=arctanImUOx,y,dReUOx,y,d

(15) ϕRx,y,d=arctanImURx,y,dReURx,y,d

Here, *ϕ*_*O*_(*x*, *y*, *d*) and *ϕ*_*R*_(*x*, *y*, *d*) represent the phases with and without the object information on the image plane, respectively. Therefore, the phase difference between them can be given by Equation 16:

(16) $$\begin{array}{ll} \Delta \phi \left(x,y,d\right) & =\phi_{O}\left(x,y,d\right)-\phi_{R}\left(x,y,d\right)\;\left(\mathrm{if}\;\phi_{O}\rangle \phi_{R}\right)\\ & =\phi_{R}\left(x,y,d\right)-\phi_{O}\left(x,y,d\right)+2\pi \;(\mathrm{if}\;\phi_{O}\langle \phi_{R}) \end{array}$$

The optical path length should be considered by the phase difference in order to obtain the 3-D metrological information of the target object. The optical path length can be obtained from the phase difference, the wave number, and the refractive index in the target object, which is given by Equation 17:

(17) $$\Delta \phi \left(x,y,d\right)=\frac{2\pi }{\lambda }\Delta n\left(x,y,\;d\right)\Delta L\left(x,y,\;d\right)$$

where Δ*n*(*x*, *y*, *d*) and Δ*L*(*x*, *y*, *d*) represent the changes of the refractive index and the thickness, respectively. Finally, the shape of the target object can be quantitatively obtained as follows:

(18) $$\Delta L\left(x,\;y,\;d\right)=\frac{\lambda }{2\pi }\frac{\Delta \phi \left(x,\;y,\;d\right)}{\Delta n\left(x,\;y,\;d\right)}$$

## Results and discussion

### Experiments

Figure 3 shows an optical setup of the proposed DHM system based on a modified LSI. In the experiment, a random linearly polarized He-Ne laser with the continuous wave (CW) output power of 2 mW at 632.8 nm is used as a light source, which has been collimated in the experiments. A transparent touch-glass panel with defects of scratches and digs whose sizes ranged from 100 to 180 μm is used as the test object. The optical beam from the He-Ne laser passes through the test object and magnifies with an objective lens (×20, NA = 0.40). Here, the hologram pattern is generated by controlling the sizes of the object beams which are reflected from the front and back surfaces of the optical window glass. The window glass plate used in the experiment has a dimension of 100 mm × 100 mm × 12 mm. For efficient interference of two object beams, the LSD is optimized by combined use of the incident beam angle and the thickness of the optical window glass.

**Figure 7:**
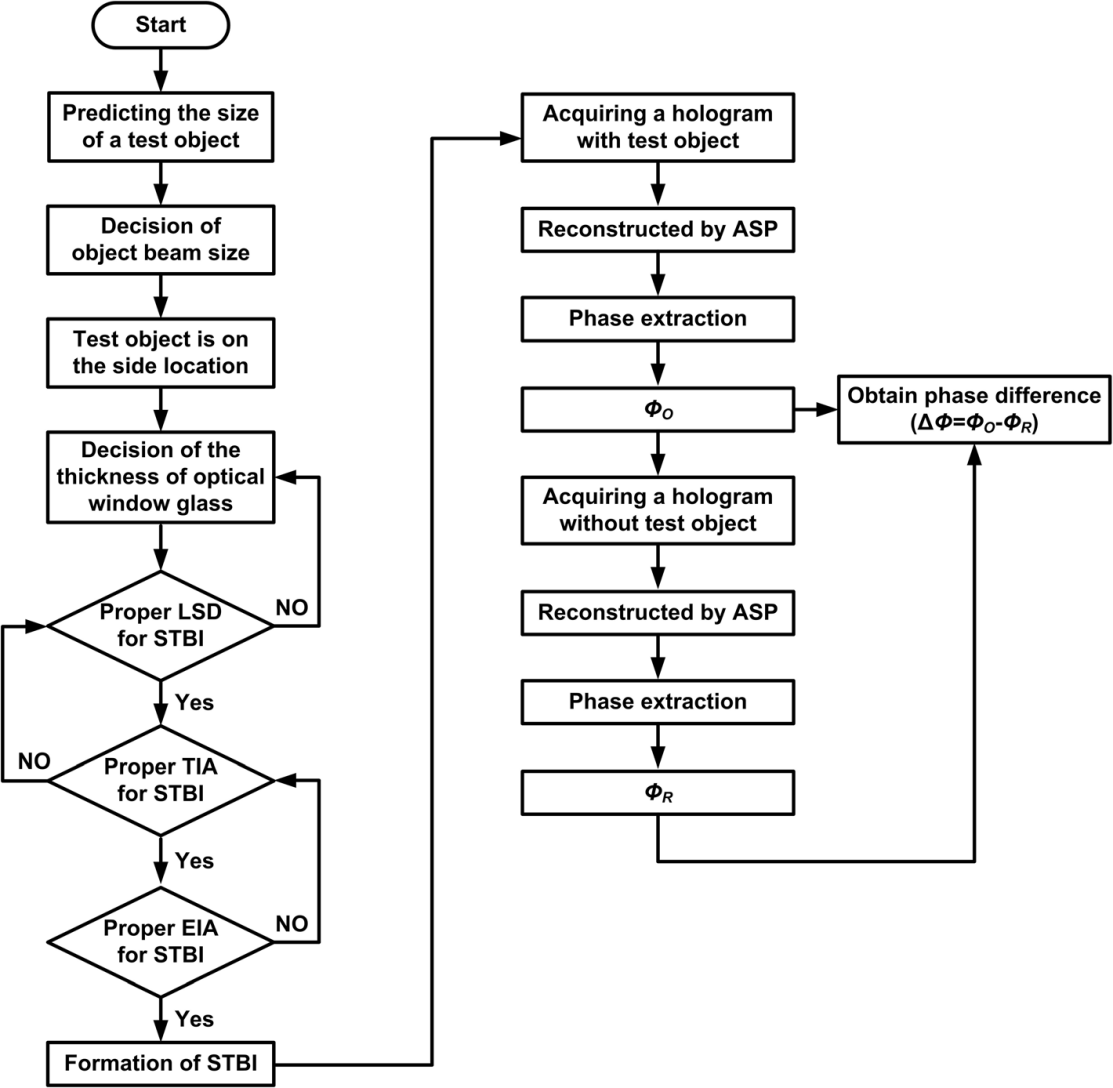
Operational flowchart of the proposed system.

Here, the thickness of the optical window glass *t*, the radius of the object beam *r*, and the incident beam angle *θ*_*i*_ are set to be 12 mm, 5 mm, and 19°, respectively. With these parameters, the LSD is controlled to be 5 mm. Also, the hologram pattern is recorded using the CCD camera with 8-bit dynamic range and 1,392 × 1,036 pixels, where each pixel has a resolution of 4.65 μm *×* 4.65 μm.Figure 7 shows an overall operational flowchart of the proposed system. Initially, the size of an object beam and the thickness of a window glass have been decided. And then, the optimized values of the LSD, TIA, and EIA are derived. Under this condition, the hologram patterns generated by the STBI concept are obtained and from which the depth data of the defects are digitally extracted.

### Experimental results

To confirm the feasibility of the proposed method, experiments with a test touch-glass panel with defects of scratches and digs are carried out. For detection of scratches and digs of the test object, hologram patterns of two sheared object beams of the test object are captured by the CCD camera using a × 20 objective lens. Then, 3-D images are reconstructed from these recorded holograms, and the results are compared to those of the conventional LSI system.

#### Detection of scratches on the test touch-glass panel

**Figure 8:**
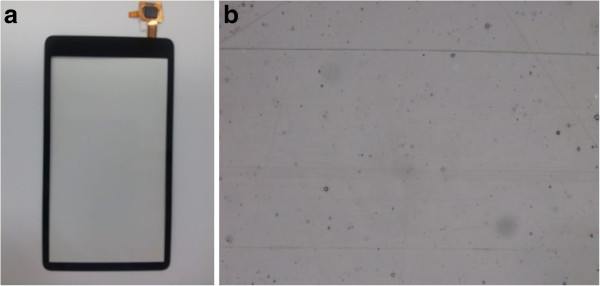
**External view and its magnified image of the test touch-glass panel. (a)** External view and **(b)** magnified image (×10).

First, scratches on the test object are detected with the conventional LSI system as well as the proposed system for comparison. Figure 8a,b shows the external view of the test object and its magnified image with the 2-D optical microscope with a × 10 objective lens (BX-51-P, OLYMPUS Corp., Tokyo, Japan).

##### Imaging of micrometer-scale scratches with the conventional LSI system

**Figure 9:**
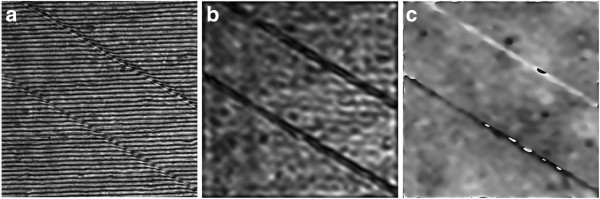
**Experimental results of the conventional system. (a)** Recorded hologram, **(b)** reconstructed intensity image, and **(c)** reconstructed phase image.

In the conventional LSI system, the thickness of the optical window glass and the object beam's radius are set to be 3 and 5 mm, respectively. Under this condition, the LSD and the TIA are calculated to be in the ranges of 0 to 2.3 mm and 76° to 90°, respectively, which means that the EIA may not be optimally formed. Thus, duplicate images may be contained in the recorded hologram and two kinds of line-scratch images, which are referred to real and duplicate images, are reconstructed from this hologram. Figure 9a is the recorded hologram pattern of a line scratch of the test object showing two line-scratch images. Intensity and phase images reconstructed from this recorded hologram are also shown in Figure 9b,c, respectively.

**Figure 10:**
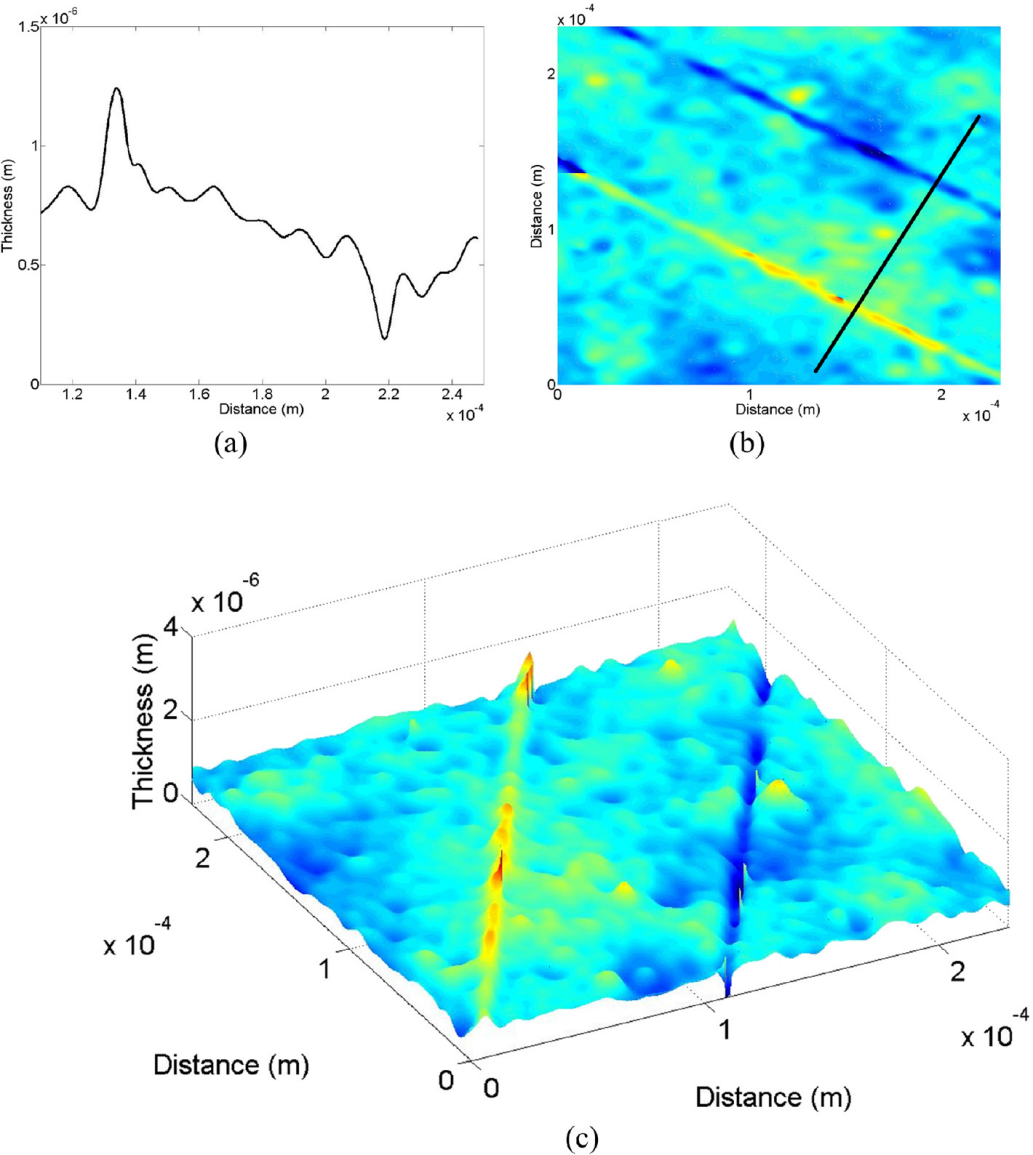
**Reconstructed line-scratch images of the touch-glass panel of the conventional system. (a)** 1-D image, **(b)** 2-D image, and **(c)** 3-D image.

From the reconstructed phase image, the depth data of the line scratch can be numerically reconstructed, and its 1-D, 2-D, and 3-D versions are shown in Figure 10. Figure 10c represents the 3-D image of the reconstructed line scratch showing a pair of line scratches. Here, the right one refers to the real image, while the left one refers to the duplicate image which is reversed in phase as seen in Figure 10a. For both of the real and duplicate images, the depth and width data of the line scratch are estimated to be about *-*380 nm and 3 μm, and 340 nm and 3 μm, respectively*.* As seen in Figure 10, here in the conventional LSI system, only part of the reconstructed image can be used for the real image, and an additional segmentation process of the real image from the duplicate image is needed for data extraction. Moreover, as the defect gets larger, the reconstructed real and duplicate images get closer and begin to overlap together. Under this condition, depth and width data of the defect cannot be effectively extracted, which means that the conventional LSI system is limited to detection of the single defect with a very small dimension.

##### Imaging of micrometer-scale scratches with the proposed system

**Figure 11:**
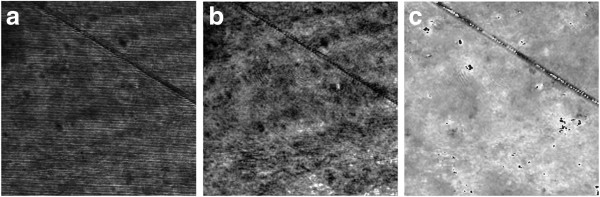
**Experimental results of the proposed system. (a)** Recorded hologram, **(b)** reconstructed intensity image, and **(c)** reconstructed phase image.

In the proposed system, the thickness of the optical window glass and the object beam's radius are set to be 12 and 5 mm, respectively. Thus, the ranges of the LSD and the TIA are calculated to be in 0 to 9 mm and 23° to 90°, respectively. In the experiment, the TIA and the incident beam angle are set to be 60° and 19°, respectively. Under this condition, duplicate images may not be contained in the recorded hologram. Figure 11 shows the recorded hologram of the line scratch of the test object. Figure 11b,c also shows the intensity and phase images reconstructed from this recorded hologram, respectively, in which only one line scratch referred to the real image is reconstructed without its duplicate version.

**Figure 12:**
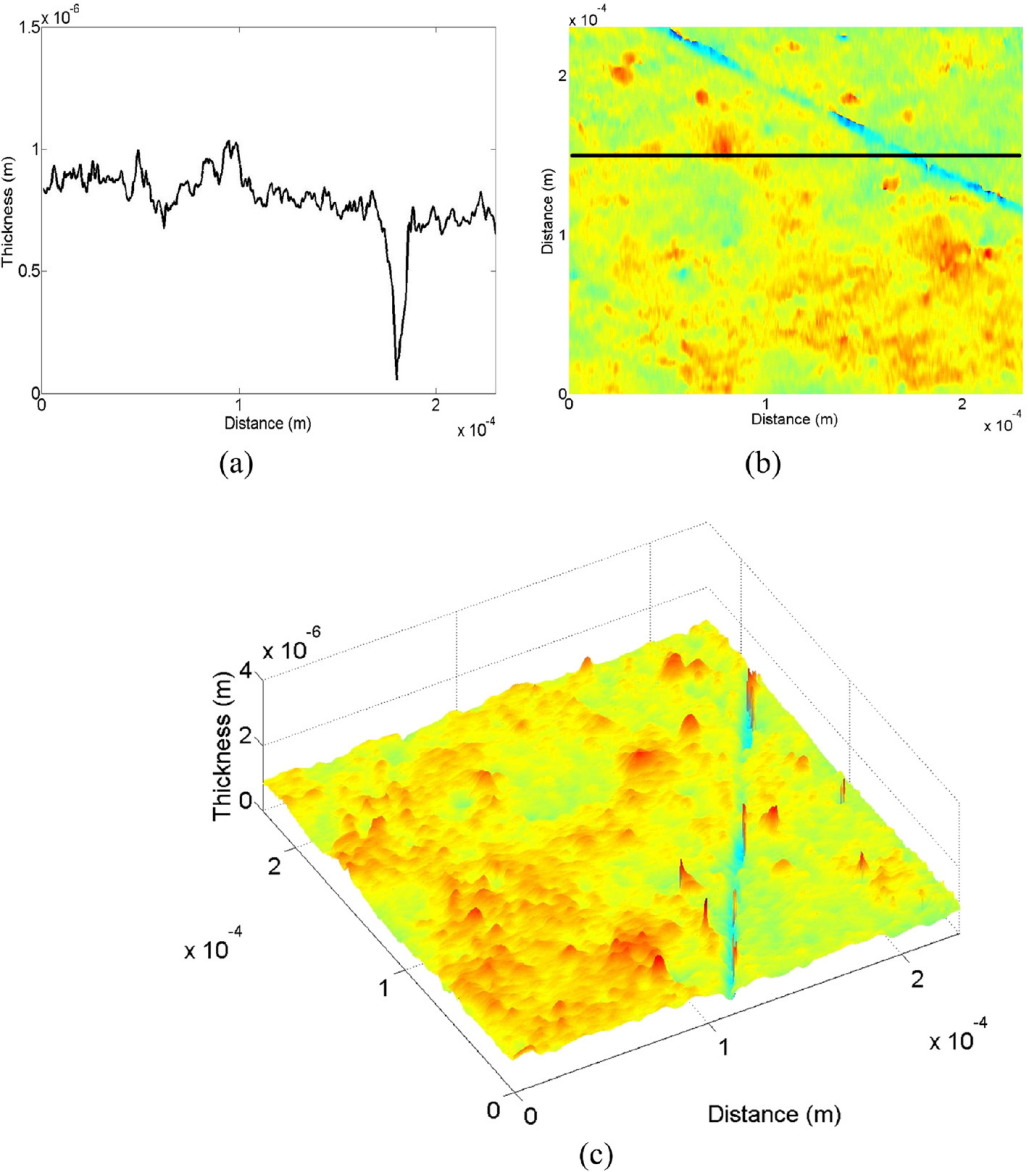
**Reconstructed line-scratch images of the touch-glass panel of the proposed system. (a)** 1-D image, **(b)** 2-D image, and **(c)** 3-D image.

From the phase image of Figure 11c, depth data of the line scratch is numerically reconstructed. The 1-D, 2-D, and 3-D versions of the depth image are shown in Figure 12. As mentioned above, only the single line-scratch image is reconstructed in the proposed system unlike the conventional LSI system. For the real image, the depth and width data of the line scratch are estimated to be about -390 nm and 3 μm, respectively, on the average. Here, it is found that there is a 10 nm difference between two depth data estimated by the conventional and proposed systems for the real image. This means that the real image may be affected by the duplicated image in the conventional LSI system. Moreover, the proposed system can utilize the full size of the reconstructed image for the real image without a need of a segmentation process, and it can be applied to detection of multi-defects with large dimensions unlike the conventional LSI system.

#### Detection of digs on the touch-glass panel

Digs as well as scratches on a touch-glass panel make errors in the production process of smart phones. Thus, detection of dig defects is very important for discrimination of high-quality panels compared to those of low quality. Here, digs on the test object are also detected by both the conventional and proposed LSI systems. Moreover, for their performance comparison, a dig with a relatively large dimension is detected.

##### Imaging of micrometer-scale digs with the conventional LSI system

**Figure 13:**
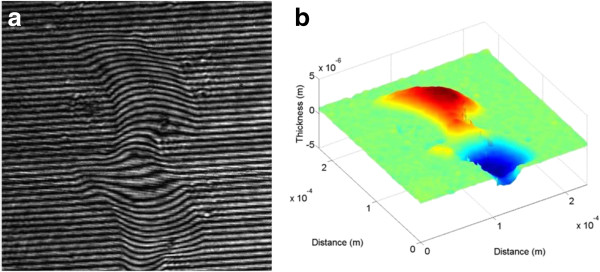
**Reconstructed real dig image of the touch-glass panel of the conventional system. (a)** Recorded hologram and **(b)** 3-D reconstructed image.

**Figure 14:**
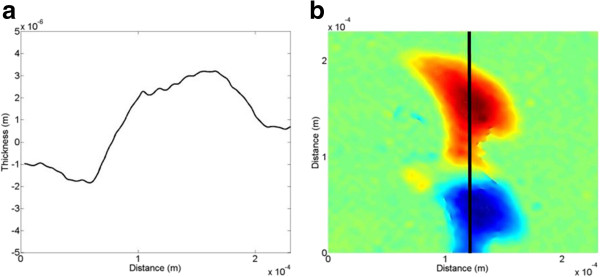
**Reconstructed dig image of the touch-glass panel of the conventional system. (a)** 1-D image and **(b)** 2-D image.

Just like the line-scratch detection, two kinds of dig images, which are real and duplicate images, are reconstructed from the recorded hologram. Figure 13a,b shows the recorded hologram and the reconstructed 3-D image of a random-shaped dig on the test panel, respectively. As seen in Figure 13b, two images are partially overlapped and mixed up since the size of the dig defect under detection is much larger than that of the line scratch. Thus, from this real image, which has been greatly influenced by the closely located duplicate image, the depth and width data of the dig cannot be detected in the conventional LSI system. Figure 14a,b shows the 1-D and 2-D versions of the reconstructed dig image.

As mentioned above, in the conventional system, the thickness of the optical window glass and the object beam's radius are set to be 3 and 5 mm, respectively. Under this circumstance, the LSD and the TIA are calculated to be 100 μm and 89.47°, respectively, which means that the effective interference area cannot be optimally formed. In other words, the shearing distance of two object beams seems very small compared to the dig having a size of about 100 to 180 μm; thus, real and duplicated images cannot be separated. Even though the depth data of the dig is estimated to be about 2.52 μm from the mixed image, this value may not be valid data for the real depth of the dig. Hence, the dig's surface area cannot be detected because two real and duplicate images have been partially overlapped to each other as shown in Figure 14b. Thus, the conventional LSI system has a limitation in application to detection of relatively large defects since the EIA cannot be sufficiently formed for the interference of two object beams without duplicate images.

##### Imaging of micrometer-scale digs with the proposed system

**Figure 15:**
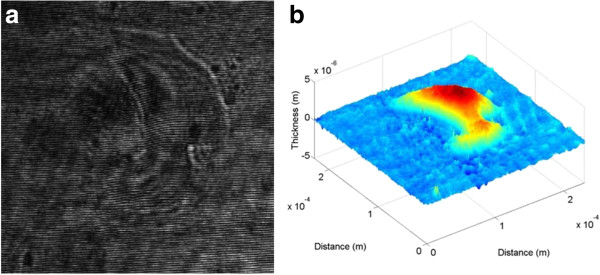
**Reconstructed dig images of the touch-glass panel of the proposed system. (a)** Recorded hologram and its **(b)** 3-D image.

**Figure 16:**
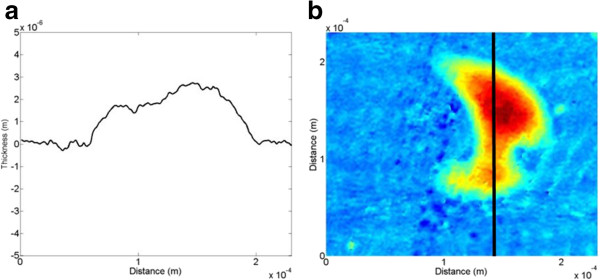
**Reconstructed dig images of the touch-glass panel of the proposed system. (a)** 1-D image and **(b)** 2-D image.

For the efficient formation of the EIA, in the proposed system, the LSD and the TIA are set to be 5 mm and 19°, respectively, under the condition that the thickness of the optical window glass and the radius of the object beam are 12 and 5 mm, respectively, as mentioned above. Using the phase image reconstructed from the recorded hologram of Figure 15a, the depth data of the dig on the test touch-glass panel is numerically reconstructed, which is shown in Figure 15b as a 3-D form. In addition, its 1-D and 2-D versions are also displayed in Figure 16a,b, respectively. Just like the line-scratch detection, only one dig image, which is referred to the real image, is reconstructed in the proposed system, which means that the duplicate image has been clearly removed from the reconstructed images.

From a real image, the depth value of the dig is estimated to be 1.45 μm on the average, and its surface area is also measured to be about 180 μm × 100 μm. Here, it is found that there exists a 1.07 μm difference between two estimated depth data of the conventional and the proposed systems, but it does not mean anything because two real and duplicate images have been mixed up together. In other words, the real image has been heavily influenced by the duplicate image in the reconstructed process in the conventional LSI system. On the other hand, in the proposed method, only the real image has been reconstructed without the duplicate image; therefore, exact detection of depth and shape data of the dig can be possible even though the dimension of dig relatively looks large.

## Conclusions

In this paper, a new type of the DHM system based on a modified LSI has been proposed for detection of micrometer- or nanometer-scale defects on transparent target objects. In the proposed system, the so-called duplicate image problem of the conventional LSI has been alleviated by employing a new concept of STBI. It means that the proposed system can operate just like most conventional two-beam interferometer systems. Successful experiments with the test object of a touch-glass panel for mobile displays confirm the feasibility of the proposed method and the possibility of its practical application to three-dimensional visual inspection of micrometer- or nanometer-scale defects on transparent objects by taking advantage of its simple optical configuration as well as its two-beam interferometer operation.

## Competing interests

The authors declare that they have no competing interests.

## Authors’ contributions

KBS did the experiments and wrote the paper. BMK participated in the discussion of this study. ESK conceived the study and revised the paper. All authors read and approved the final manuscript.
